# Combination Therapy With Rapamycin and Low Dose Imatinib in Pulmonary Hypertension

**DOI:** 10.3389/fphar.2021.758763

**Published:** 2021-11-11

**Authors:** Yinan Shi, Chenxin Gu, Tongtong Zhao, Yangfan Jia, Changlei Bao, Ang Luo, Qiang Guo, Ying Han, Jian Wang, Stephen M. Black, Ankit A. Desai, Haiyang Tang

**Affiliations:** ^1^ College of Veterinary Medicine, Northwest A&F University, Yangling, China; ^2^ Department of Medicine, Krannert Institute of Cardiology, Indiana University, Indianapolis, IN, United States; ^3^ State Key Laboratory of Respiratory Disease, National Clinical Research Center for Respiratory Disease, Guangdong Key Laboratory of Vascular Disease, Guangzhou Institute of Respiratory Health, The First Affiliated Hospital of Guangzhou Medical University, Guangzhou, China; ^4^ Department of Critical Care Medicine, Suzhou Dushu Lake Hospital, The First Affiliated Hospital of Soochow University, Suzhou, China; ^5^ Department of Physiology, Nanjing Medical University, Nanjing, China; ^6^ Department of Cellular Biology and Pharmacology, Herbert Wertheim College of Medicine, Miami, FL, United States; ^7^ Department of Environmental Health Sciences, Center for Translational Science, Robert Stempel College of Public Health and Social Work, Florida International University, Port St. Lucie, FL, United States

**Keywords:** MTOR signaling, PDGFRs, PAH, PASMCs, rapamycin, imatinib

## Abstract

**Rationale:** Enhanced proliferation and distal migration of human pulmonary arterial smooth muscle cells (hPASMCs) both contribute to the progressive increases in pulmonary vascular remodeling and resistance in pulmonary arterial hypertension (PAH). Our previous studies revealed that Rictor deletion, to disrupt mTOR Complex 2 (mTORC2), over longer periods result in a paradoxical rise in platelet-derived growth factor receptor (PDGFR) expression in PASMCs. Thus, the purpose of this study was to evaluate the role of combination therapy targeting both mTOR signaling with PDGFR inhibition to attenuate the development and progression of PAH.

**Methods and Results:** Immunoblotting analyses revealed that short-term exposure to rapamycin (6h) significantly reduced phosphorylation of p70S6K (mTORC1-specific) in hPASMCs but had no effect on the phosphorylation of AKT (p-AKT S473, considered mTORC2-specific). In contrast, longer rapamycin exposure (>24 h), resulted in differential AKT (T308) and AKT (S473) phosphorylation with increases in phosphorylation of AKT at T308 and decreased phosphorylation at S473. Phosphorylation of both PDGFRα and PDGFRβ was increased in hPASMCs after treatment with rapamycin for 48 and 72 h. Based on co-immunoprecipitation studies, longer exposure to rapamycin (24–72 h) significantly inhibited the binding of mTOR to Rictor, mechanistically suggesting mTORC2 inhibition by rapamycin. Combined exposure of rapamycin with the PDGFR inhibitor, imatinib significantly reduced the proliferation and migration of hPASMCs compared to either agent alone. Pre-clinical studies validated increased therapeutic efficacy of rapamycin combined with imatinib in attenuating PAH over either drug alone. Specifically, combination therapy further attenuated the development of monocrotaline (MCT)- or Hypoxia/Sugen-induced pulmonary hypertension (PH) in rats as demonstrated by further reductions in the Fulton index, right ventricular systolic pressure (RVSP), pulmonary vascular wall thickness and vessel muscularization, and decreased proliferating cell nuclear antigen (PCNA) staining in PASMCs.

**Conclusion:** Prolonged rapamycin treatment activates PDGFR signaling, in part, via mTORC2 inhibition. Combination therapy with rapamycin and imatinib may be a more effective strategy for the treatment of PAH.

## Introduction

Pulmonary arterial hypertension (PAH) is characterized by progressive increases in pulmonary vascular resistance (PVR) and pressure, which can lead to deterioration of right ventricular function (Voelkel et al., 2012; Humbert et al., 2019). The major factors contributing to the elevated PVR include sustained increases in pulmonary vascular contraction and obliterative pulmonary vascular remodeling (Rabinovitch, 2008; Archer et al., 2010; Schermuly et al., 2011). Similar to the pathways in cancer, hyperproliferation and enhanced migration of PASMCs are considered as major contributors to the vascular remodeling (Guignabert et al., 2013). Molecular mechanisms of PAH are complex and involve multiple signaling pathways including cell survival and proliferation, such as phosphoinositide 3-kinase (PI3K)/AKT/mammalian target of rapamycin (mTOR), platelet-derived growth factor (PDGF), and transforming growth factor (TGF)-β/bone morphogenetic protein (BMP) (Rabinovitch, 2008; Tang et al., 2018a; Rol et al., 2018).

mTOR is a key serine/threonine protein kinase that responds to a variety of extracellular stimuli including growth factors, insulin, nutrients (amino acids, glucose), and hypoxic stress (Lin et al., 2017). mTOR functions as the key component of two similar protein complexes: mTOR complex l (mTORC1) and mTORC2. mTORC1 regulates ribosomal S6 protein kinase (p70S6K) and eukaryotic promoter 4e binding protein 1 (4E-BP1), which both play important roles in protein translation and cell growth, respectively. mTORC2 controls cell growth, apoptosis, and regulates tumorigenesis through phosphorylation of AKT (Gulati et al., 2009; Jhanwar-Uniyal et al., 2017). The mTOR pathway is a major research focus for a variety of diseases including an ongoing clinical trial with the mTOR inhibitor, rapamycin in PAH (Houssaini et al., 2013; Arriola Apelo and Lamming, 2016).

Prior studies have shown that mTORC1 inhibition with rapamycin attenuates pre-clinical PAH development (Houssaini et al., 2013; Goncharova et al., 2020). Furthermore, deletion of tuberous sclerosis complex ½ (TSC 1/2), which is an upstream inhibitor of mTORC1 signaling, also inhibits expression of PDGFRs in a rapamycin-sensitive manner (Zhang et al., 2007). In contrast to these data, smooth muscle cell-specific ablation of mTORC2 over prolonged periods results in spontaneous murine PAH, possibly due to unexpected increases in PDGFR expression (Tang et al., 2015; Tang et al., 2018a). Cumulatively, these findings raise concerns for a paradoxical increased risk in PDGFR activation with prolonged therapeutic use of rapamycin in PAH.

Receptor tyrosine kinase (RTK) signaling, including PDGF, mediates lung vascular remodeling in PAH (Perros et al., 2008a). RTK signaling activates several proliferative signaling cascades including PI3K, phospholipase Cγ (PLcγ), Ras-mitogen-activated protein kinase (Ras-MAPK) and Janus kinase (JAK) (Hubbard and Till, 2000). Imatinib, a tyrosine kinase inhibitor, decreases autophosphorylation of PDGFRs, resulting in the inhibition of PDGF signaling. Despite results of Phase II study demonstrating reasonable tolerability of imatinib in patients with PAH, severe adverse events, significant side effects, and a high discontinuation rate appeared in the Phase III trial, which clearly show that higher doses of imatinib are not suitable and limit the utility of imatinib in the treatment of PAH (Ghofrani et al., 2010; Frost et al., 2015). Moreover, additional studies further demonstrated that low dose imatinib did not attenuate the development of experiment PH, highlighting the importance of targeting other molecular pathways in PAH (Schermuly et al., 2005). While mTOR and PDGFR signaling pathways both independently hold potential for targeting as therapeutics in PAH, the effects of prolonged exposure to rapamycin on the activity of PDGFRs and PH development remain unknown. Thus, the aim of this study was to dissect mechanisms of short-term and prolonged rapamycin use on PDGFR signaling during PAH treatment and to investigate the therapeutic potential of combined therapy with rapamycin and the RTK inhibitor, imatinib.

## Materials and Methods

### Animal Model and Experimental Design.

All animals in the studies were handled according to the National Institutes of Health guidelines and approved by the Institutional Animal Care and Use Committee (IACUC) of Guangzhou Medical University. For the MCT model, male Sprague-Dawley (SD) rats (200–250 g) were studied after a single intraperitoneal monocrotaline (MCT) injection (50 mg/kg, #HY-N0750, MCE, United States). For the hypoxia-SU5416 model, SD rats (200–250 g) placed under hypoxic condition (FiO_2_ 10%) for 3 weeks after given single dose of SU5416 (20 mg/kg, #HY-10374, MCE, United States). They were transitioned to normoxia for 2 weeks. Rats were randomly assigned to four groups: rapamycin alone (2 mg/kg/d, #HY-10219, MCE, United States), imatinib alone (10 mg/kg/d, #S1026, Selleck, United States), rapamycin (2 mg/kg/d) combined with imatinib (10 mg/kg/d), or vehicle only. All drugs were given once daily by intraperitoneal injection for the last 2 weeks of each model. Terminal measurements were recorded. Right ventricular systolic pressure (RVSP) was measured as previously published (Tang et al., 2018b) with a pressure transducer catheter (Millar Instruments) and used as a surrogate for pulmonary arterial pressure. Hearts were excised and dissected to determine the ratio of the RV weight to the left ventricle (LV) and septum (S) weight ratio.

### Cell Culture and Experiments

Human PASMCs (hPASMCs) were obtained from Sciencell (#3110) and Promocell (#399Z003.1). Cells were maintained in SMC medium (#1101, Sciencell, United States of America) with 2% fetal bovine serum and SMC growth supplement (#1152, Sciencell, United States). For migration assay, hPASMCs suspension was diluted to 2 × 10^5^/ml. 70 ul of cell suspension was seeded into culture-inserts of 2-wells in 35 mm dish (#81176, IBIDI, Germany). After cell attachment, inserts were removed using sterilized forceps. Cells were cultured in serum-free medium containing rapamycin (100 nM), imatinib (5 μM) or vehicle and pictures recorded every 4 h. The number of cells migrating out of the wound edge was scored using Image J image analysis software. Cell viability was assessed by CCK8 kit (#96992, Sigma-Aldrich, United States). hPASMCs were seeded into 96-well plates at a density of 5,000 cells per well with six replicates per group. Cells were cultured in 2% serum-containing medium exposed to either rapamycin (100 nM), imatinib (5 μM), or vehicle. After addition of 10ul CCK8 per well, the plate was cultured for 4 h at 37°C. The absorbance value of each well was measured and collected at 450 nm. Cell proliferation assays were performed using BrdU incorporation kit (#QIA58, Merck Millipore, United States). hPASMCs were seeded into 96-well plates at a density of 5,000 cells per well with four replicates per group. Cells were cultured in 2% serum-containing medium exposed to either rapamycin (100 nM), imatinib (5 μM), or vehicle. BrdU assay was conducted according to the standard protocol of the manufacturer and BrdU uptake was measured in each well at dual wavelengths of 450–540 nm.

### Western Blot and Co-Immunoprecipitation

Total cellular and isolated pulmonary arterial (PA) proteins were extracted using RIPA lysis buffer containing proteinase and phosphatase inhibitor. Western blotting was performed using the following antibodies: anti-mTOR (#2983), anti-Rictor (#5379) anti-phospho-PDGFRα/β (#3170), anti-PDGFRα (#3174), anti-PDGFRβ (#3169), anti-phospho-AKT (T308) (#13038), anti-phospho-AKT (S473) (#4060), anti-AKT (Pan) (#4691), anti-phospho-p70S6k (#9204), anti-p70S6k (9202), anti-phospho-S6 (#4858), and anti-S6 (#2317) (Cell Signaling Technology, United States). For Co-immunoprecipitation, cell protein was extracted in the non-deformed protein lysate. Non-specific proteins were removed by adding protein A/G-beads (#sc-2003, Santa Cruz Biotechnzazology, United States). An antibody targeting mTOR was incubated at room temperature to bind the antibody to the target antigen. The antigen-antibody complex was incubated overnight at 4°C and then, boiled for 5 mins. SDS-polyacrylamide gel electrophoresis was performed by adding the sample buffer on the protein (Sarbassov et al., 2006).

### Immunofluorescence

For immunofluorescence staining, lung tissue sections were blocked in 2% BSA for 30 min and incubated overnight at 4°C with alpha-smooth muscle actin (α-SMA) and proliferating cell nuclear antigen (PCNA). After extensive washes with PBS for 30 min, lung tissue was incubated with Alexa 488 anti-rabbit secondary or Alexa 594 anti-mouse secondary antibodies at a 1:500 dilution for 1 h at room temperature. Nuclei were stained with DAPI (4, 6-diamidino-2-phenylindole).

### Statistical Analysis

Data were presented as means ± standard error (SE) and analyzed by GraphPad Prism software. Statistical differences among multiple experimental groups were tested by one-way analysis of variance (ANOVA) and two-way ANOVA. *p* < 0.05 was considered as statistically significant.

## Results

### Rapamycin Inhibits Both mTORC1 and mTORC2 in hPASMCs

**FIGURE 1:**
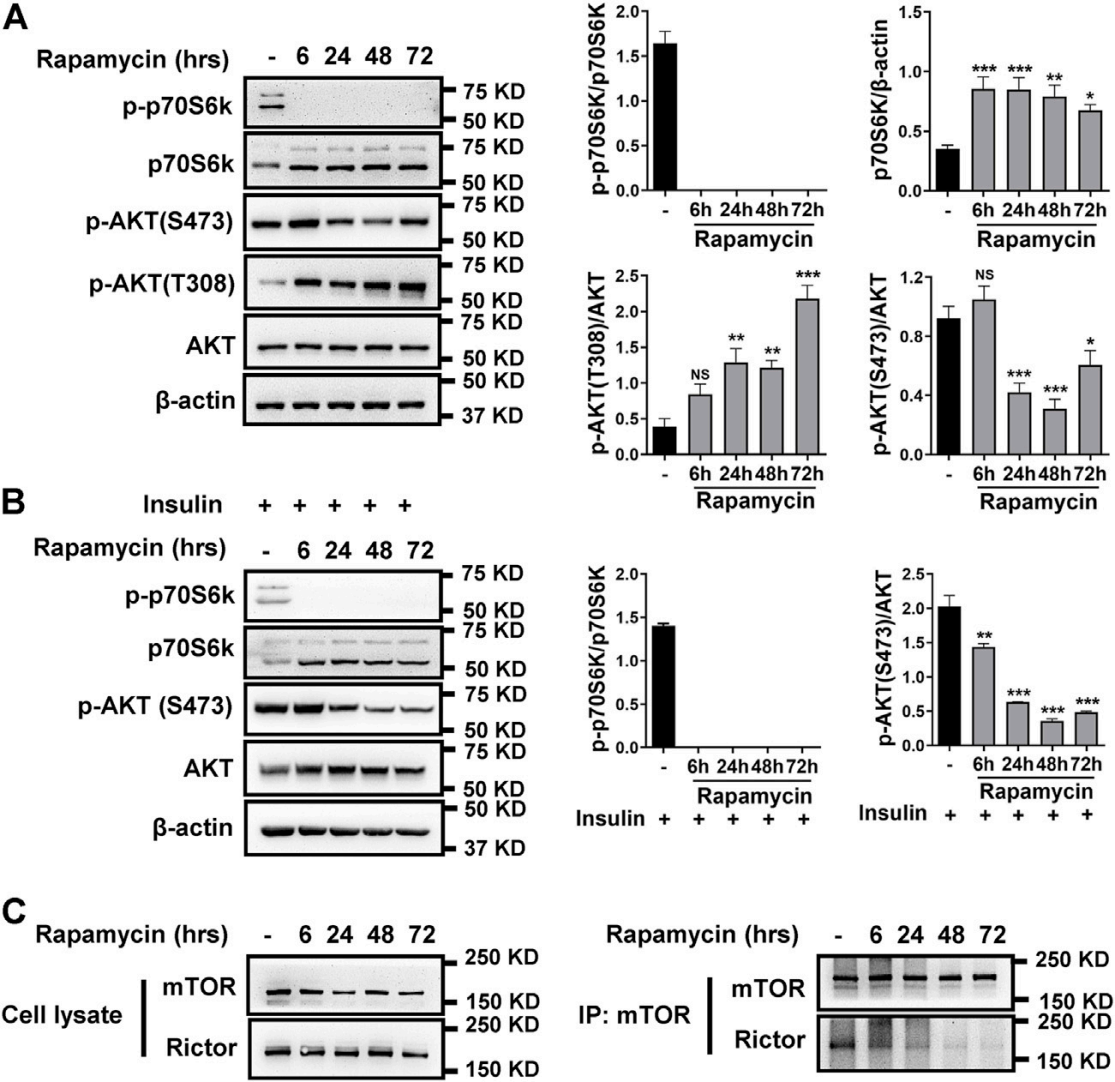
Rapamycin inhibits mTORC1 and mTORC2. **(A)** hPASMCs were treated with 100 nM rapamycin for the indicated times and analyzed by immunoblotting for the proteins level of p-p70S6k, p70S6k, p-AKT (S473), p-AKT (T308), AKT. **(B)** immunoblotting analyses of p-p70S6k, p70S6k, p-AKT (S473), and AKT in hPASMCs, which were stimulated with 5 μg/ml insulin for 24h before treatment with 100 nM rapamycin. **(C)** hPASMCs were treated with 100 nM rapamycin for the indicated times, and then cell lysates were prepared for and immunoprecipitation (IP) with mTOR antibody. The elution from IP was analyzed by immunoblotting for the levels of mTOR and Rictor. Data are presented as the mean ± SE. One-way ANOVA was used for statistical analysis. NS means not significant. ***p < 0.001; **p < 0.01; *p < 0.05 versus control.

While rapamycin is well-established as an mTORC1 inhibitor, its effects on mTORC2 has not been well studied. Thus, we initially investigated the effects of longer rapamycin exposure on mTORC2 in hPASMCs. As shown in Figure 1A, rapamycin treatment reduced phosphorylated p70S6k (p-p70S6k) and increased total p70S6k levels across all time points in hPASMCs, validating rapamycin-mediated inhibition of mTORC1. In contrast, phosphorylated AKT (p-AKT S473) levels were reduced only with longer exposures of rapamycin, suggesting reductions of mTORC2 activity with chronic exposure. Specifically, inhibition of mTORC1 by rapamycin can result in increased availability of free mTOR generating increases in mTORC2 activity (Hussain et al., 2013). To test this possibility, hPASMCs treated with insulin to activate mTORC2 and found that p-AKT S473 was not increased after treatment with rapamycin for 6 h (Figure 1B).

Rapamycin works through a gain-of-function mechanism in which it binds to the intracellular protein FKBP12 to generate a drug-receptor complex that binds to and inhibits mTORC1 (Annett et al., 2020). To determine whether rapamycin inhibits mTORC2 activity via a similar mechanism, we evaluated the effect of rapamycin on the interaction between Rictor and mTOR in hPASMCs using immunoprecipitation analysis. As shown in Figure 1C, rapamycin caused a time-dependent reduction in the binding between Rictor and mTOR. Expression levels of mTOR and Rictor remained unchanged in whole cell lysates.

### Rapamycin Treatment Activates PDGFRα/β

**FIGURE 2:**
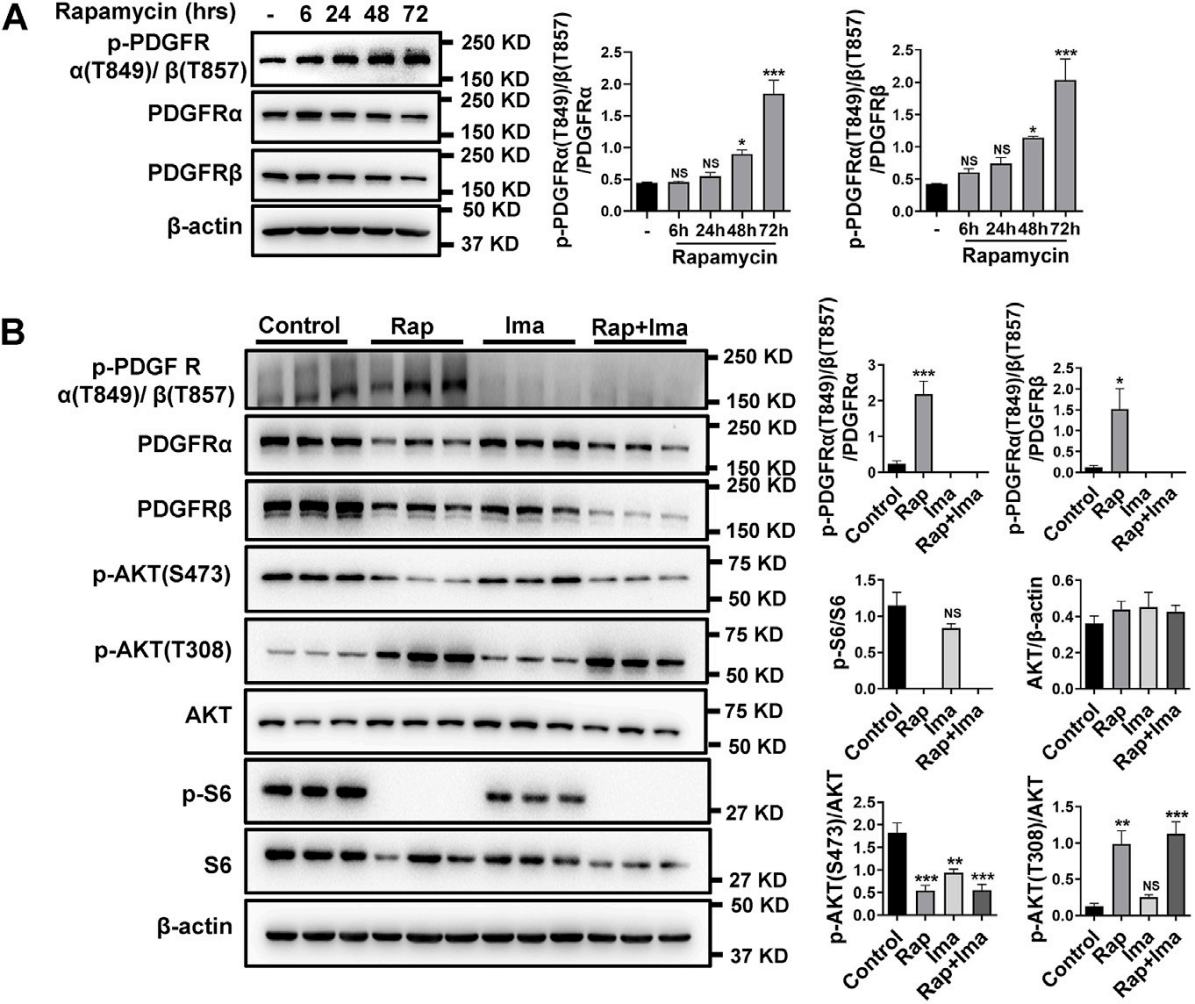
Imatinib inhibits phosphorylation of PDGFRα/β induced by rapamycin in hPASMCs. **(A)** hPASMCs were treated with 100 nM rapamycin for the indicated times and analyzed by immunoblotting for the proteins level of p-PDGFRα/β, PDGFRα, PDGFRβ. **(B)** Immunoblotting analyses of p-PDGFRα/β, PDGFRα, PDGFRβ, p-AKT (S473), p-AKT (T308), p-S6 and S6 in hPASMCs treated with vehicle (Control), 100 nM rapamycin (Rap), 5 uM imatinib (Ima) and 100 nM rapamycin + 5 uM imatinib (Rap + Ima) for 48 h. Data are presented as the mean ± SE. One-way ANOVA was used for statistical analysis. NS means not significant. ***p < 0.001, **p < 0.01, *p < 0.05 versus control.

Previously, we found elevated expression of PDGFRs in isolated pulmonary arteries (PA) from smooth muscle-specific Rictor knockout mice (*Rictor* ^SM-/-^) (Tang et al., 2018a). Base on rapamycin reducing mTORC2 activity, the effects of long-term rapamycin treatment on both phosphorylated and total PDGFRs was investigated. As shown in Figure 2A, phosphorylated PDGFR levels were increased in hPASMCs after treatment with rapamycin at 48 and 72 h. Impact of combination therapy on mTOR and PDGFR signaling pathways also was detected. Consistent with previous results, exposure to rapamycin alone significantly reduced p-S473AKT and increased phosphorylation of p-T308AKT (Figure 2B). In parallel, rapamycin increased p-PDGFRα (T849)/β (T857) in hPASMCs. Imatinib alone inhibited the phosphorylation of PDGFR. Imatinib use alone also mildly inhibited mTOR activation. Rapamycin combined with imatinib robustly inhibited the activation of mTORC1 and mTORC2 along with the phosphorylation of PDGFRα/β.

### Rapamycin Combined With Imatinib Inhibits Viability, Proliferation and Migration of hPASMCs

**FIGURE 3:**
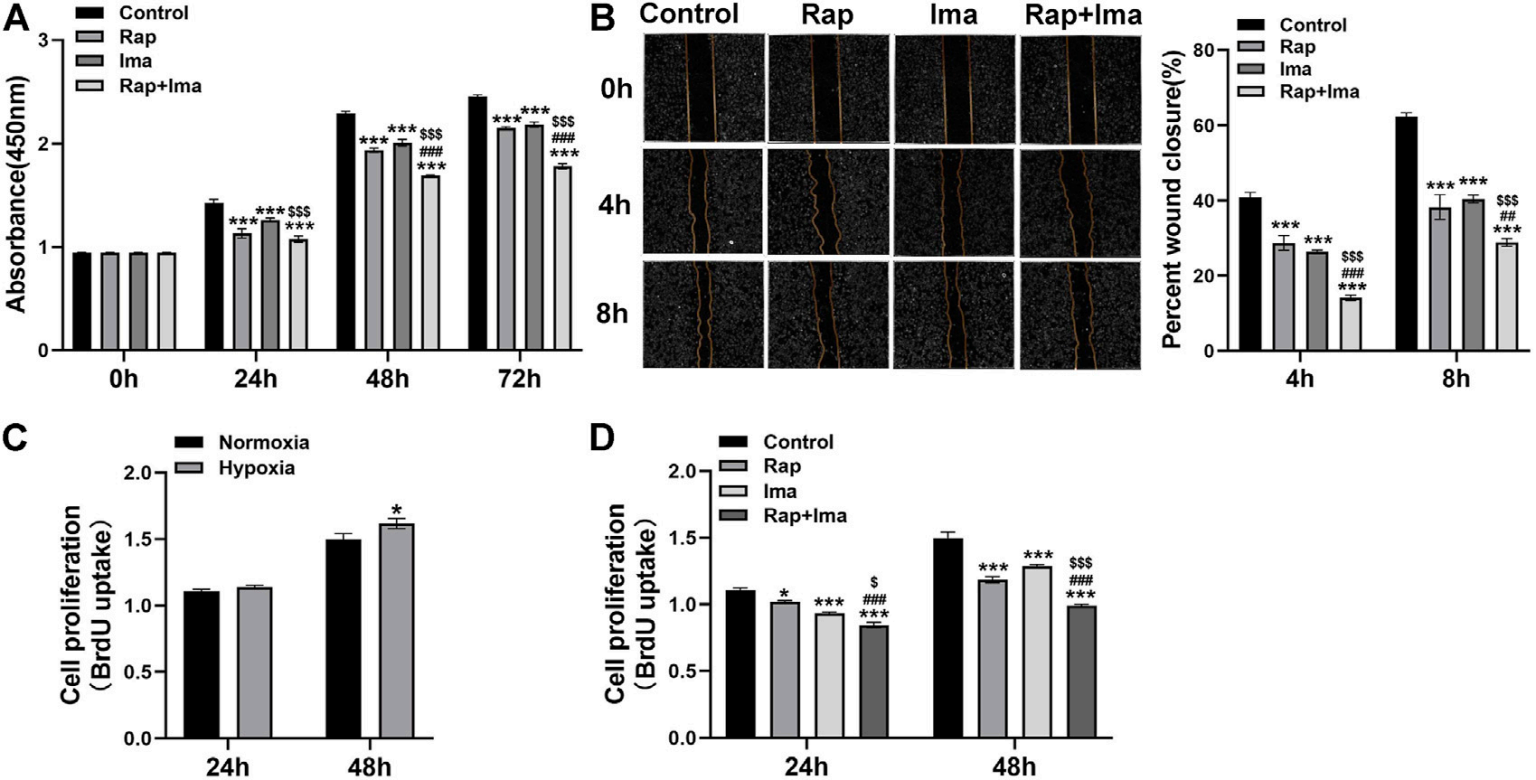
Effects of rapamycin combined with imatinib on the viability, proliferation and migration of hPASMCs. **(A)** Cell viability was determined by measuring the absorbance at 0, 24, 48 and 72 h after different drug treatments. **(B)** A scratch was applied to cell monolayers, and migration of the cells towards the wound was recorded by photomicrographs at 0, 4, and 8h (*n* = 3); summarized data showing percent wound closure [(0h wound area–4h or 8h wound area)/0h wound area] * 100%. **(C)** BrdU assay was performed to determine hPASMCs proliferation under normoxia and hypoxia (3% 0_2_) for 24 and 48 h. **(D)** BrdU assay was performed to determine hPASMCs proliferation at 24 and 48 h after different drug treatments. Data are presented as the mean ± SE. Two-way ANOVA was used for statistical analysis. ***p < 0.001; **p < 0.01; *p < 0.05 versus control; ^**###**^ *p* < 0.001, ^**##**^ *p* < 0.01, ^**#**^ *p* < 0.05 versus Rap; ^**$$$**^ *p* < 0.001, ^**$$**^ *p* < 0.01, ^**$**^ *p* < 0.05 versus Ima.

Since hPASMCs proliferation and migration drives obliterative pulmonary arterial vascular remodeling in PAH, we investigated the impact of rapamycin and imatinib on each process. We first detected impact of rapamycin and imatinib on cell viability. Result showed that both rapamycin and imatinib alone significantly inhibited hPASMCs viability. Importantly, the inhibitory effects of rapamycin combined with imatinib was more significant than that of either drug alone (Figure 3A). Both drugs alone significantly inhibited the migration of hPASMCs using a cell scratch assay, while the inhibitory effect of the combined regimen was more potent than either drug alone (Figure 3B). To further confirm the inhibitory effects of rapamycin combined with imatinib on PASMCs proliferation, a BrdU assay was performed. As shown in Figure 3C, hypoxia induced hPASMCs proliferation at 48 h, validating our prior observations (Tang et al., 2015; Tang et al., 2016). Both rapamycin and imatinib alone significantly inhibited hPASMCs proliferation (Figure 3D) despite hypoxic exposure. The inhibitory effect of the combined regimen was more potent than either drug alone.

### Rapamycin Combined with Imatinib Attenuates PH in MCT and Hypoxia/Sugen Treated Rats

**FIGURE 4:**
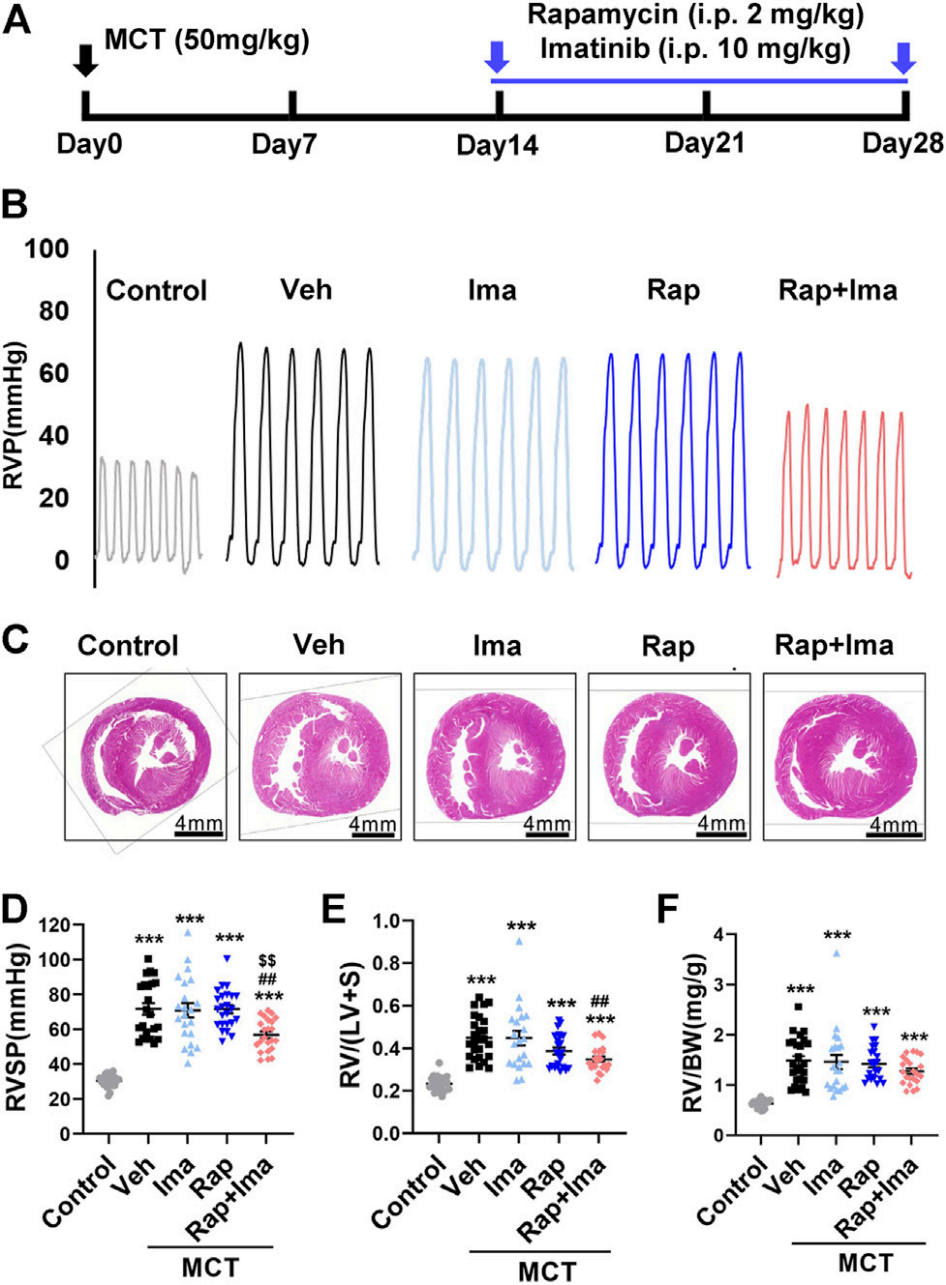
Rapamycin combined with imatinib attenuates PH induced by MCT. **(A)** Schematic strategy for the MCT-induced PH rodent model and drugs administration. **(B)** Representative record of right ventricle pressure (RVP) in control and MCT rats treated with vehicle (Veh), imatinib (Ima), rapamycin (Rap), or combination of rapamycin and imatinib (Rap + Ima). **(C)** H&E staining of heart tissue in each group. **(D,E,F)** Summarized data showing RVSP, Fulton index ([RV/(LV + S)]) and the ratio of right ventricle weight to body weight (RV/BW) respectively. Data are presented as the mean ± SE. One-way ANOVA was used for statistical analysis. ***p < 0.001, **p < 0.01, *p < 0.05 versus control; ^**###**^ *p* < 0.001, ^**##**^ *p* < 0.01, ^**#**^ *p* < 0.05 versus MCT with vehicle; ^**$$$**^ *p* < 0.001, ^**$$**^ *p* < 0.01, ^**$**^ *p* < 0.05 versus MCT with rapamycin.

We evaluated combination therapy with rapamycin and imatinib in MCT and Hypoxia/Sugen rat models of PAH. Rats develop PH in both MCT and Hypoxia/Sugen treated (Figures 4A, 6A), as evidenced by significant increases in RVSP, [RV/(LV + S)] and RV/BW (Figures 4B–F, 6B–E) compared to normal control animals. In the MCT model, as shown in Figures 4B–F, neither rapamycin or low dose imatinib (10 mg/kg) alone not attenuated the development of PH in MCT-rats with no significant reductions in RVSP, the Fulton index and RV/BW, compared to controls. However, combination therapy significantly decreased RVSP and Fulton index, but no effect on RV/BW. In the Hypoxia/Sugen rat model, as shown in Figures 6B–E, rapamycin exposure alone resulted in significant decreases in RVSP and the Fulton index but not RV/BW. Imatinib alone decreased the Fulton index but had no effect on RVSP and RV/BW. However, combination therapy resulted in significantly decreased RVSP, Fulton index and RV/BW, compared to controls.

**FIGURE 5:**
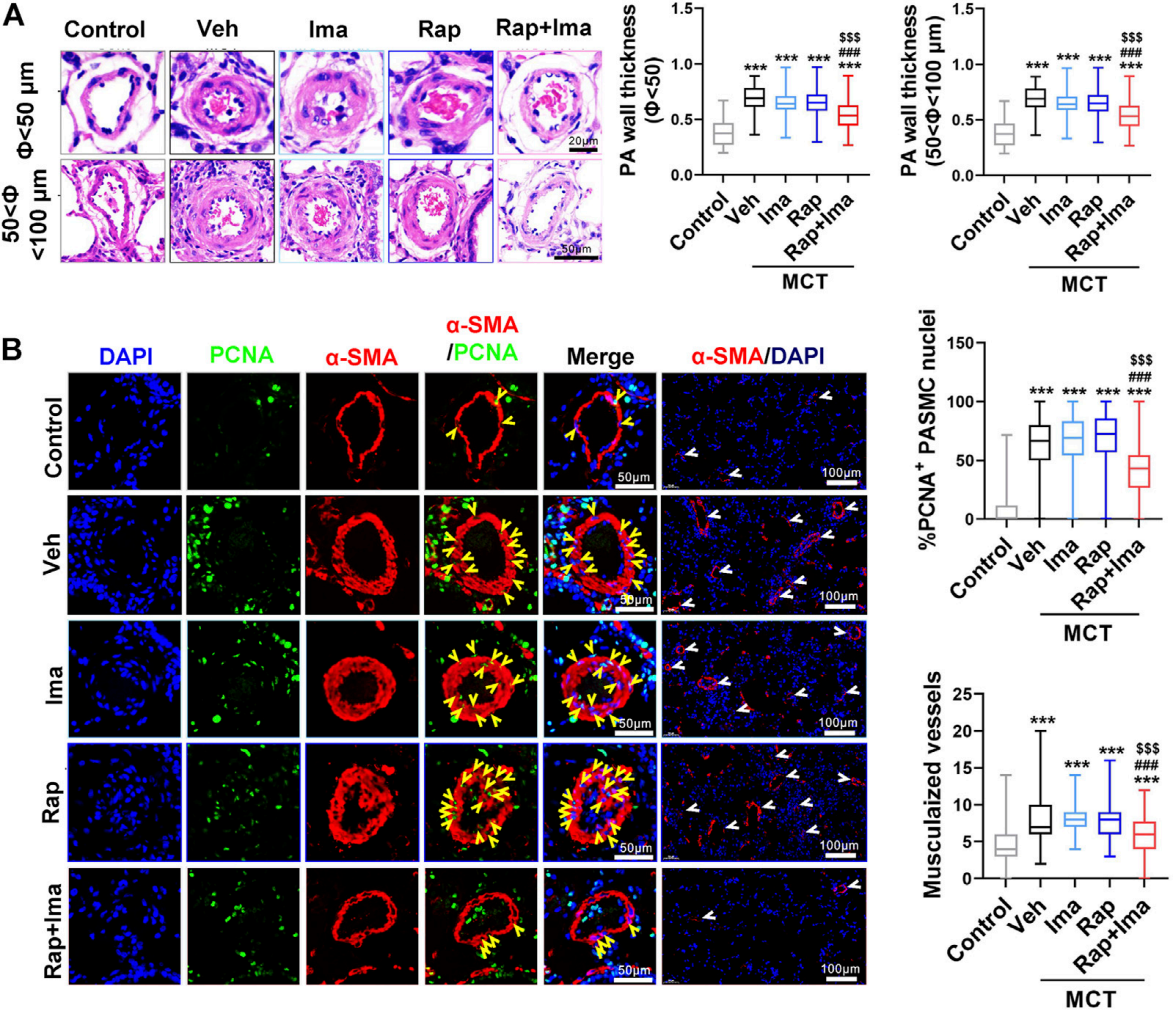
Rapamycin combined with imatinib attenuates PASMC proliferation and remodeling induced by MCT. **(A)** H&E staining in lung tissue sections. Summarized data showing pulmonary artery media wall thickness. **(B)** Lung sections were stained α-SMA (red) and PCNA (green). Yellow arrowheads point at PCNA positive PASMCs and white arrowheads show the vessels. For each of the 5 groups, approximately 600 PASMC nuclei and 100 fields were analyzed. Summarized data showing PCNA positive cells and muscularization. Data are presented as the mean ± SE. One-way ANOVA was used for statistical analysis. ***p < 0.001, **p < 0.01, *p < 0.05 versus control; ^**###**^ *p* < 0.001, ^**##**^ *p* < 0.01, ^**#**^ *p* < 0.05 versus MCT with vehicle; ^**$$$**^ *p* < 0.001, ^**$$**^ *p* < 0.01, ^**$**^ *p* < 0.05 versus MCT with rapamycin.

We next evaluated the effects of combination therapy on the extensive vascular remodeling and muscularized vessels in both rat models. For the MCT rodent model (Figures 5A,B), H&E staining showed that rapamycin or imatinib alone had no significant effect on pulmonary vascular wall thickening or the number of muscularized vessels. However, combination therapy significantly inhibited the thickening of the vascular wall and the number of muscularized vessels. Moreover, rapamycin or imatinib alone had no effect on the percentage of proliferating cell nuclear antigen (PCNA)-positive PASMCs in the lung. The combination strategy significantly reduced the percentage of PCNA-positive PASMCs in small pulmonary arterioles. Similarly, in the Hypoxia/Sugen model (Figures 7A,B), imatinib alone had no significant effect on pulmonary vascular thickening or the number of muscularized vessel. Rapamycin alone significantly inhibited the thickening of pulmonary vascular thickening with diameter less than 50 μm but had no effect on the number of muscularized vessels. Combination therapy of rapamycin with imatinib significantly inhibited both of vascular wall thickening and the number of muscularized vessels. Additionally, PCNA staining revealed significantly decreased cell proliferation within small pulmonary arterioles after combination strategy.

**FIGURE 6:**
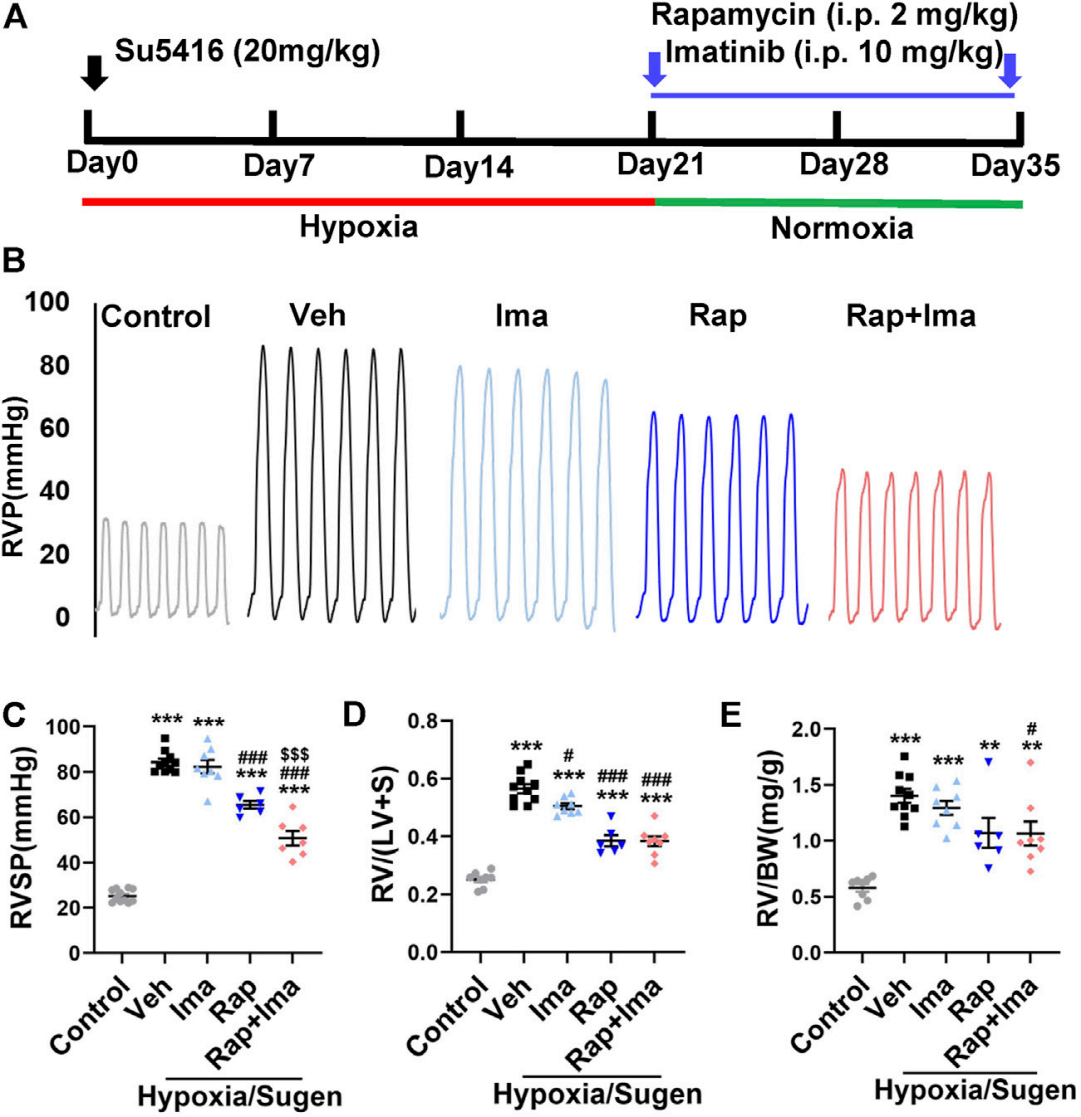
Rapamycin combined with imatinib attenuates PH induced by Hypoxia/Sugen. **(A)** Schematic strategy for the Hypoxia/Sugen-induced PH rodent model and drugs administration. **(B)** Representative record of right ventricle pressure (RVP) in control and Hypoxia/Sugen treated with vehicle (Veh), imatinib (Ima), rapamycin (Rap) or combination of rapamycin and imatinib (Rap + Ima). **(C,D,E)** Summarized data showing RVSP, Fulton index [RV/(LV + S)] and the ratio of right ventricle weight to body weight (RV/BW) respectively. Data are presented as the mean ± SE. One-way ANOVA was used for statistical analysis. ***p < 0.001, **p < 0.01, *p < 0.05 versus control; ^**###**^ *p* < 0.001, ^**##**^ *p* < 0.01, ^**#**^ *p* < 0.05 versus Hypoxia/Sugen with vehicle; ^**$$$**^ *p* < 0.001, ^**$$**^ *p* < 0.01, ^**$**^ *p* < 0.05 versus Hypoxia/Sugen with rapamycin.

**FIGURE 7:**
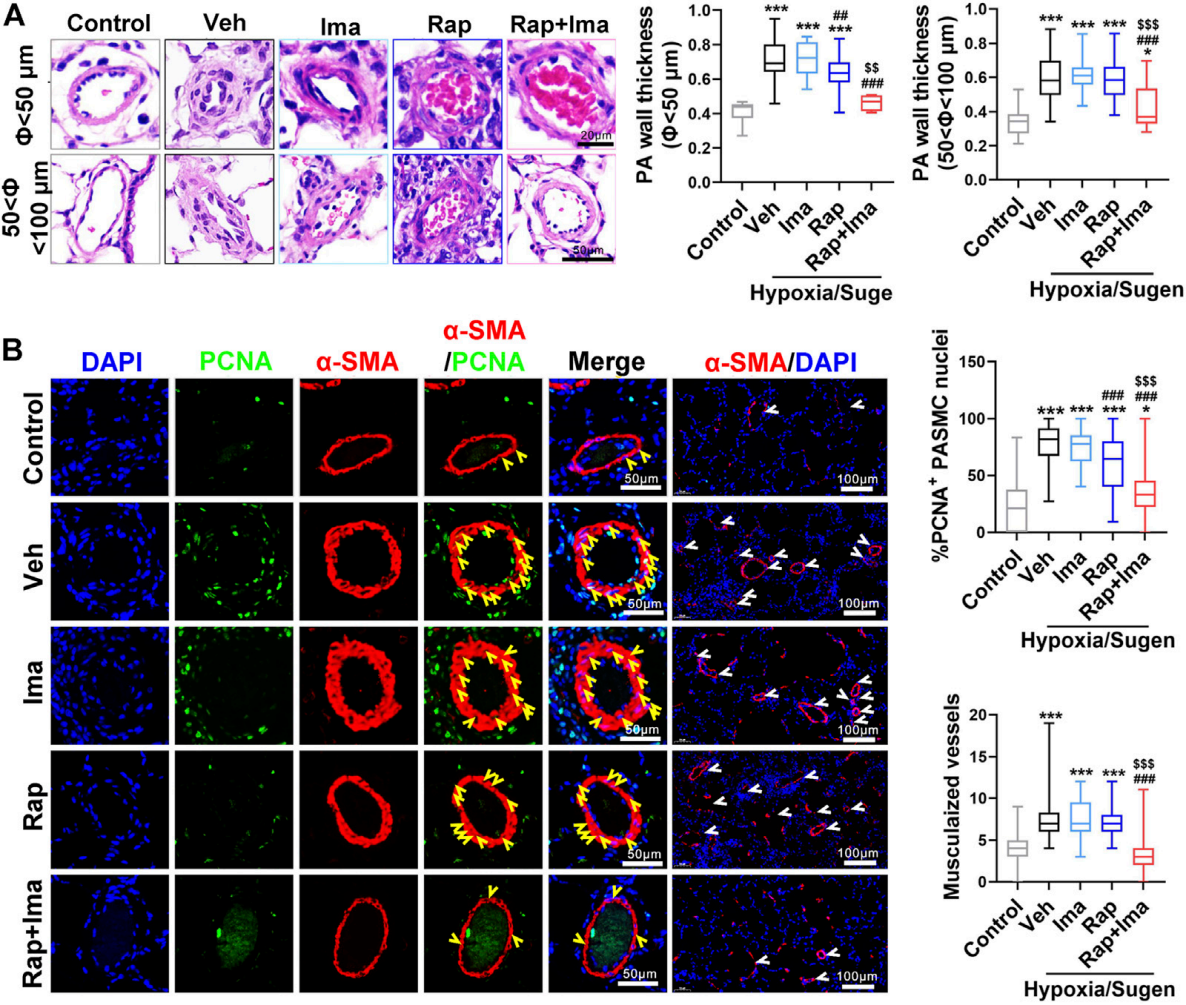
Rapamycin combined with imatinib attenuates PASMC proliferation and remodeling induced by Hypoxia/Sugen. **(A)** H&E staining of lung tissue sections and summarized data showing pulmonary artery media wall thickness. **(B)** Lung sections were stained with α-SMA (red) and PCNA (green). Yellow arrowheads point at PCNA positive PASMCs and white arrowheads show the vessels. For each of the 5 groups, approximately 600 PASMC nuclei and 100 fields were analyzed. Summarized data showing PCNA positive cells and muscularization. Data are presented as the mean ± SE. One-way ANOVA was used for statistical analysis. NS means no significant. ***p < 0.001, **p < 0.01, *p < 0.05 versus control; ^**###**^ *p* < 0.001, ^**##**^ *p* < 0.01, ^**#**^ *p* < 0.05 versus Hypoxia/Sugen with vehicle; ^**$$$**^ *p* < 0.001, ^**$$**^ *p* < 0.01, ^**$**^ *p* < 0.05 versus Hypoxia/Sugen with rapamycin.

### Rapamycin Combined With Imatinib Inhibits mTOR and PDGFR Signaling Pathway in Isolated Pulmonary Arteries

**FIGURE 8:**
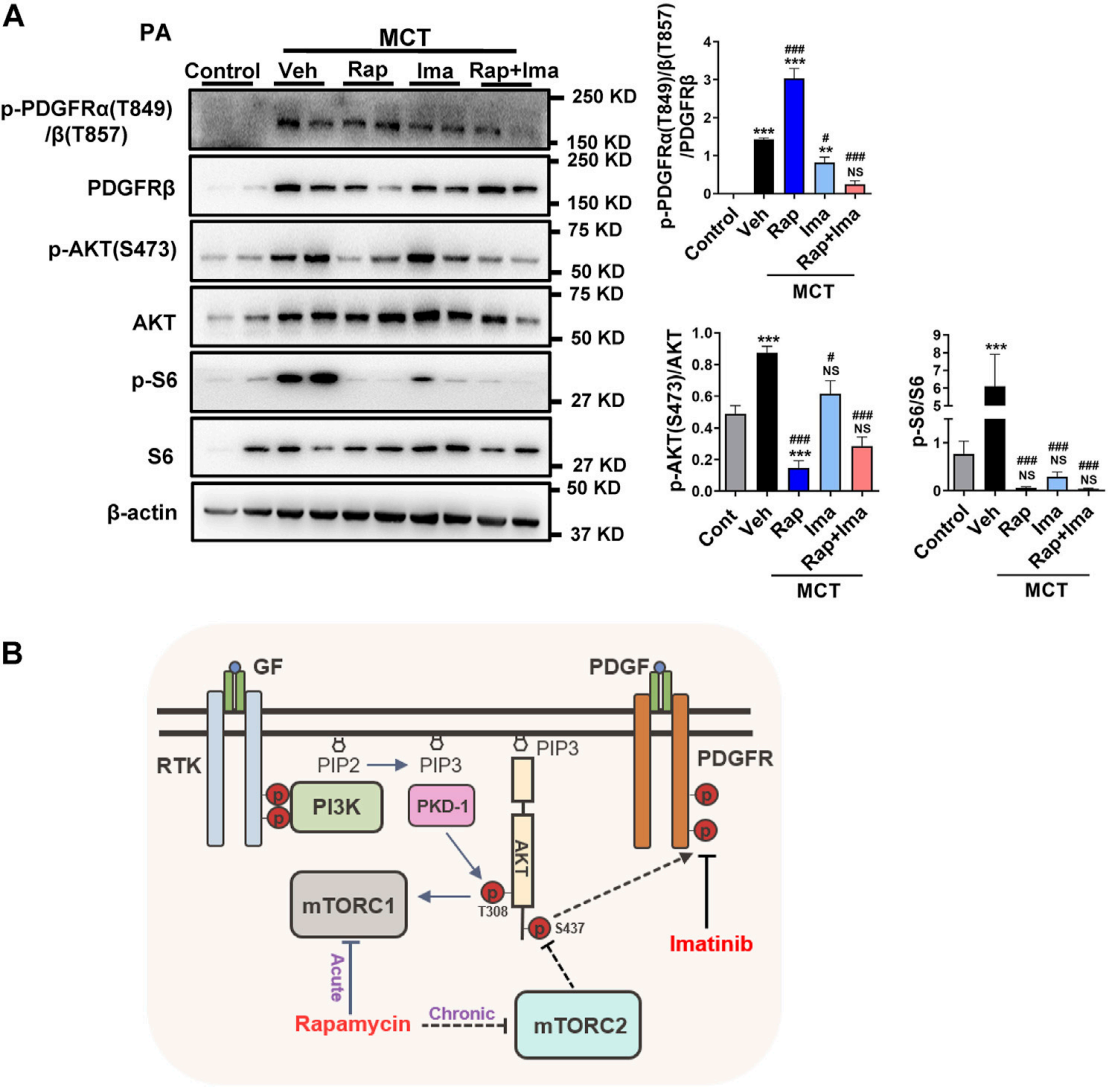
Effects of rapamycin combined with imatinib on mTOR and PDGFR signaling pathways in pulmonary artery. **(A)** Pulmonary artery vessels of were isolated for protein extraction, and the expression of mTORC 1, mTORC 2 and PDGFR signaling pathway related proteins were detected by immunoblotting. Data are presented as the mean ± SE. One-way ANOVA followed by Graphpad prism was used for statistical analysis. NS means no significant. ***p < 0.001; **p < 0.01; *p < 0.05 versus control. ^**###**^ *p* < 0.001; ^**##**^ *p* < 0.01; ^**#**^ *p* < 0.05 versus MCT with vehicle. **(B)** The schematic representation of the findings of this study: rapamycin chronic treatment in hPASMCs induced the highly expression of phosphorylation of PDGFRs. Imatinib inhibits phosphorylation of PDGFRα/β induced by rapamycin. Abbreviations: GF, growth factors; RTK, receptor tyrosine kinase; PDGF, platelet derived growth factor; PDGFR, platelet derived growth factor receptor; PI3K, phosphoatidylinositol 3-kinase; PIP2, phosphatidylinositol-4,5-bisphosphate; PIP3, phosphatidylinositol-3,4,5-bisphosphate; mTORC1, mTOR complex 1; mTORC2, mTOR complex 2.

We evaluated the effects of combination therapy on mTOR and PDGFR signaling pathways in isolated PAs from rat lungs. As shown in Figure 8A, expression of p-PDGFR, p-S6 and pS473AKT was increased in PAs isolated from MCT-exposed rats. Confirming *in vitro* data, rapamycin inhibited the activity of both, mTORC1 and mTORC2. Imatinib alone inhibited the phosphorylation of PDGFR. Rapamycin combined with imatinib inhibited mTORC1, mTORC2 and PDGFR signaling mediators.

## Discussion

Both rapamycin and imatinib have been tested in clinical trials in patients with PAH (Ghofrani et al., 2005; Wessler et al., 2010). While the results of the latter have been unfavorable, the results of the trial testing rapamycin remain pending. On the surface, rapamycin and imatinib appear to inhibit two unrelated molecular targets including mTORC1 and PDGFR, respectively. Building upon our prior observations, our studies uncover a crossover between the two signaling cascades that could be leveraged to potentially develop a novel combination therapeutic strategy. Specifically, the current study confirms findings of a paradoxical and an unexpected rise in PDGFR activation with prolonged exposure to rapamycin *in vitro* and *in vivo*. While mTORC1 is a known target, our data suggest the PDGFR activation is due, in part, to additional inhibition of mTORC2 by rapamycin. Devising a strategy to address both seemingly related pathological cascades, the current study found greater attenuation of PH in two pre-clinical rat models when rapamycin and imatinib were combined compared to either drug alone.

The PI3K/AKT/mTOR signaling pathway mediates PAH pathogenesis via its canonical role in cell survival and proliferation (Li et al., 2007; Tang et al., 2015). Rapamycin was approved by the US Food and Drug Administration as an immunosuppressant and has been proved to significantly inhibit mTORC1. However, the effect of rapamycin on mTORC2 is controversial (Sarbassov et al., 2004; Sarbassov et al., 2006; Schreiber et al., 2015). In fact, targeting this pathway for drug development requires a better understanding of the complexity of the cascade. Briefly, AKT is normally maintained in an inactive situation. However, AKT was activated by binding of phosphatidylinositol-3,4,5-bisphosphate (PIP3) generated by PI3K phosphorylated phosphatidylinositol-4,5-bisphosphate (PIP2), within biological membranes. Co-recruited Phosphoinositide-dependent protein kinase-1(PDK1) activates mTORC1 through the phosphorylation of AKT at Thr308 (Laplante and Sabatini, 2009; Hers et al., 2011), while mTORC2 phosphorylates AKT at Ser473 in a PI3 kinase-independent manner (Tsuchiya et al., 2014). Validating prior studies (Chung et al., 1992), we found that rapamycin inhibited mTORC1 with both short- and long-term exposure in hPSMCs. In addition, we observed a significant reduction in phosphorylated AKT (S473) with longer exposures of rapamycin, indicative of rapamycin-mediated mTORC2 inhibition. This observation was further supported by co-immunoprecipitation studies that demonstrated that rapamycin significantly inhibits the binding of Rictor to mTOR after 6 h, which is considered necessary to activate p-S473AKT.

Beyond reductions in p-S473AKT with longer exposures, rapamycin also inhibited p70S6k phosphorylation at 6 h, with a trend toward activation of p-S473AKT. A compensatory response by mTORC2 could explain this discrepant finding. Specifically, mTOR is a common component of the two complexes, and both mTORC1 and mTORC2 compete for mTOR binding (Hussain et al., 2013). With shorter exposures, only mTORC1 is inhibited by rapamycin, resulting in increased availability of free mTOR that may result in a secondary increase of mTORC2 activity. Consistent with this idea, when insulin, which can activate mTORC2, was added into hPASMCs, we observed no change in p-S473AKT levels at 6 h.

Bolstering findings from our previous studies in *Rictor* ^SM-/-^ mice that demonstrated increased expression of PDGFRs with aging (Tang et al., 2018a), we found that sustained exposure to rapamycin increases PDGFR activation. PDGF is expressed in various cell types such as endothelial cells, smooth muscle cells and macrophages (Perros et al., 2008b). PDGF promotes cell proliferation, migration, and cell survival by activating two subtypes of receptors, PDGFRα and PDGFRβ (Heldin and Westermark, 1999). PDGF and PDGFR are significantly upregulated in lung tissues of PAH patients and experimental animal models of PAH. Given its pathological role in PAH, PDGFR signaling has been evaluated as a therapeutic target in PAH (Barst, 2005; Perros et al., 2008a; Grimminger and Schermuly, 2010). The current study further supports the notion of targeting PDGFR for therapeutic benefit in PAH. Rapamycin alone was able to mildly attenuate pre-clinical rodent PH with longer exposures, with its therapeutic effect potentially diminished by the reciprocal activation of PDGFR signaling. Moreover, the latter observation may have been mediated, in part, by the inhibition of mTORC2 with longer rapamycin exposures. To support this notion, we observed enhanced therapeutic attenuation of pre-clinical rodent PAH when we combined rapamycin with PDGFR inhibition using imatinib. Beyond robustly improving the hemodynamics and obliterative pulmonary vascular remodeling in two established rodent models, the combination therapy was able to inhibit the compensatory activation of p-PDGFRs induced by long-term treatment of rapamycin.

In summary, in contrast to mTORC1 inhibition with shorter duration, prolonged rapamycin treatment results in the inhibition of both mTORC1 and mTORC2. As illustrated in Figure 8B, long term exposure to rapamycin also results in activation of PDGFR signaling in PASMC, that is likely due to the mTORC2 inhibition. Combination therapy with rapamycin and imatinib was a more effective strategy that either drug alone in preventing PH development, alleviating both the pulmonary vascular remodeling, and improving right heart hemodynamics. These data highlight the need to further investigate the translatability of these findings in clinical trials of both drugs in PAH.

## Data Availability

The original contributions presented in the study are included in the article/Supplementary Material, further inquiries can be directed to the corresponding authors.
